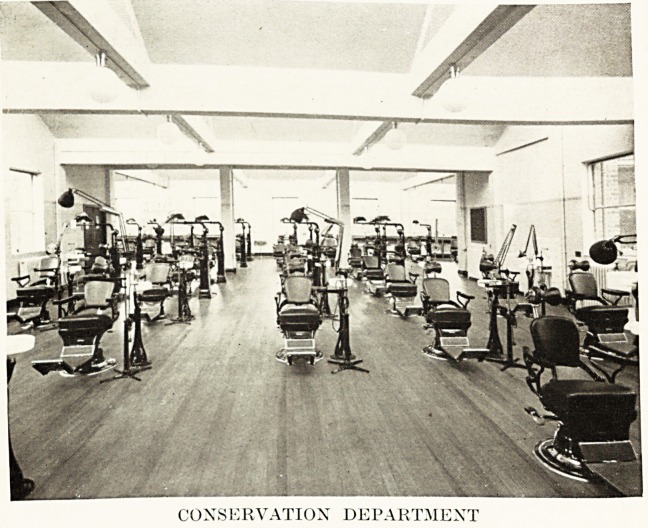# University of Bristol Dental Hospital and School

**Published:** 1940

**Authors:** 


PLATE I

UNIVERSITY OF BRISTOL DENTAL HOSPITAL

CONSERVATION DEPARTMENT

### UNIVERSITY OF BRISTOL DENTAL HOSPITAL AND SCHOOL.

Quietly and with a minimum of preliminary advertisement, the Bristol Dental Hospital has been established. The manner of its foundation is new, at any rate in Bristol, because it owes its existence entirely to the University which is responsible for the cost of erection and its future maintenance. The need for our Dental Hospital is primarily educational. For many years there has been a flourishing Dental School in Bristol, which began in the days of University College, and has thriven even more vigorously since the advent of the University. But the facilities for practical work were only exiguously furnished at the Royal Infirmary and General Hospital. Long before a Dental Board for the United Kingdom existed the Dean of the Medical School (Professor Fawcett) and Messrs. Ackland and Dowling (Dental Surgeons at the Bristol Royal Infirmary and Bristol General Hospital respectively) had realized that a Dental Hospital was essential to the satisfactory teaching of Dental students in Bristol. When the Dental Board was founded in 1920 it supported the idea with all its power, and in the end with a considerable capital grant towards the building.

Professor Fawcett describes the development of the Dental School in Bristol thus:—

When he was first appointed Professor of Anatomy there was a very small entry of dental students, not more than one or two per annum. After he became Dean of the Medical Faculty he made a practice of asking the men as soon as they qualified if they could suggest improvements in the provision of teaching. About the year 1906 the answer of the newly-qualified men was immediate and unanimous to the effect that a mechanical laboratory was needed where prosthetics could be taught properly. At that time the pupillage system was in vogue, fees of varying amounts were charged for two years' apprenticeship to private dental practitioners, and in some instances the teaching of prosthetics was very poor. The *General Medical Council recommended that the training in prosthetics should be given in an institution, and Professor Fawcett told Mr. Ackland and Mr. Dowling (Honorary Dental

* The responsibility for Dental education has now passed from the General Medical Council to the Dental Board.

Surgeons to the Bristol Royal Infirmary and Bristol General Hospital respectively) that it was proposed to make compulsory under the regulations for the Bristol B.D.S. and L.D.S. that prosthetics should be studied in the University Mechanical Laboratory or an institution recognized for the same purpose. To his gratification they at once agreed, and forthwith discontinued taking private pupils. In order that there might be no complaints from local practitioners of undue competition a fee of 100 guineas was suggested. There was some difficulty in getting the matter through Council. Mr. James Arrowsmith opposed the scheme ; he would listen to nothing, and Council rejected the proposal. Professor Fawcett writes : "I went to Arrowsmith next day and as usual got frightful abuse from him (which I knew he did not really mean). When he at last consented to listen, he said : 'Why, it is a paying proposition. I will bring it up myself at the next meeting!' He did so, and it went through."

The Mechanical Laboratory was formed, and from the start it carried out the work of the Infirmary Dental Department. About this time Professor Fawcett addressed a meeting of the Association of Dental Teachers of the North, held in Manchester, and his proposal that private pupillage should be abolished was carried unanimously. He found the Dean of the Birmingham Dental School also in favour of this policy. The outcome of these discussions was that the work of the Dental Mechanical Laboratory was placed on the same footing as that of the Dissecting Room or Physiological Laboratory, and the students were charged an annual fee. Since then the Laboratory has never looked back, and in a short time the General Hospital brought its dental work into the scheme.

At the beginning there was some difficulty in providing training in gold work, and it was complained that in this respect the University training was inferior to that given by the private dentist. The Dean of the Royal Dental Hospital in London explained how this difficulty was met in his Hospital by allowing every student to make gold plates on the cast of the mouth, after which the gold was assayed and returned to stock. This simple method was at once adopted in Bristol.

Meanwhile, Mr. Ackland and Mr. Dowling were lamenting the absence of a Dental Hospital in Bristol. Professor Fawcett brought up the matter before the University Buildings Committee, pointing out that it was a very urgent and important question. Most, if not all, of the members agreed, but there was no money at hand. But the Dental Board, recently established, receives a large income from registration fees, which allows funds to be given to Dental Schools for the provision of scholarships and buildings and the improvement of teaching.

The Dental Board inspected the teaching arrangements in Bristol, and reported that they were unsatisfactory. Thereupon the Committees of the Bristol Royal Infirmary and Bristol General Hospital agreed to send their patients to a Dental Hospital if and when established. Various suggestions for opening such a hospital were considered, amongst them one for the conversion of Canynge Hall to this purpose, which was approved by the Dental staff. To Professor Fawcett's great disappointment the situation of Canynge Hall did not meet with the approval of the Dental Board, and his retirement from the office of Dean came before he could realize his ambition and see a Dental Hospital in working order in Bristol.

It fell to the lot of Professor Brocklehurst, who succeeded him as Dean, to help in the final stages. He has summarized these stages in the opening paragraphs of the following account. Dental patients have been treated and dental students have obtained their clinical practice for many years at the Bristol Royal Infirmary and the Bristol General Hospital. While treatment has been carried out without difficulty, the arrangements for teaching have been unsatisfactory; the accommodation and facilities at the two institutions are not as good as would be expected in a single hospital, and the splitting of students and cases into two units has meant wastefulness. Moreover, students have had to carry out their mechanical work in a crowded laboratory at the University, and much time has been wasted travelling between the laboratory and the hospitals.

Following an adverse report on the conditions by representatives of the Dental Board in 1928, the University explored various possibilities, including the conversion of Canynge Hall into a Dental School, but for one reason or another all the suggestions brought forward were deemed unsatisfactory. In 1935, however, a site in Lower Maudlin Street, opposite the Royal Infirmary, fell vacant; this was purchased by the University. With the generous assistance of the Dental Board and the active interest of the Treasurer of the University, Dr. Stanley Badock, a Dental Hospital and School has been erected on this site, to the plans of Mr. Eustace H. Button, F.R.I.B.A. The building was formally opened by Professor E. L. Sheridan, M.D.S., F.R.C.S.I., Chairman of the Dental Board, on Wednesday, 10th April, 1940.

The University now has a School where all its Dental teaching can be carried out under one roof. Hither are being transferred the Dental out-patients from the Royal Infirmary and General Hospital, now free to use for other purposes the space taken up by their Dental Departments. The Dental Assistant-Dean, Mr. P. J. Stoy, has supplied the following account of the building :—

The complete Hospital, in its construction, lay-out and equipment, rivals any similar hospital in the country. Externally its brick façade harmonizes with that of the new building of the Eye Hospital.

The Hospital consists of ground, first and second floors. In general lay-out the dual purpose of the building, *i.e.* treatment of patients and training of students, was kept constantly in mind when the plans were being drafted. One main entrance serves for all, but patients are then diverted to one side, staff and students to the other. Close to the entrance is the main patients' waiting-hall ; here their cards are obtained and the new patients registered. The Almoner's Office is situated close to the waiting-hall for the convenience of the patients. Nearby also is the examination room and a surgery for extractions under local anæsthesia. With these arrangements patients requiring immediate treatment circulate only in a small area close to the entrance, and there is no necessity for them to go upstairs.

The remainder of the ground floor is occupied by a lecture theatre accommodating fifty and provided with an epidiascope and lantern, students' common rooms, a locker room where each student has his own large steel locker, lavatories, a staff common room and administrative offices.

The whole of the front of the first floor is occupied by the operating theatres and their associated waiting and recovery rooms. Routine extractions under nitrous oxide anæsthesia will be done in two adjacent inter-communicating rooms, and although it is anticipated that only one operating team will work, the use of both rooms alternately will mean that long gas lists can be handled expeditiously, but without hurry or discomfort to the patients. The windows of these theatres are specially thick to prevent noise entering from the street and disturbing the semi-conscious patient, and double doors prevent noises in the surrounding rooms and corridors from being heard.

A large theatre for operations needing longer anæsthesia has been incorporated, although it is unlikely that it will be much used until the Hospital has acquired beds of its own in an adjacent wing. The anæsthetic gases are obtained in the theatres by pipe lines from large cylinders in the basement, an economic method. All the theatres are air-conditioned.

At the back of the first floor is the laboratory for Dental Mechanics, holding fifty students, and various small laboratories for such associated procedures as polishing and vulcanizing dentures. This separation of procedures will, it is hoped, keep the atmosphere in the main laboratory healthy; in addition, there is a fan to circulate the air. The windows are of frosted glass of a type which throws the light well across the laboratory, and artificial lighting is provided at each working place. The benches have rubber mountings to reduce to a minimum the transmission of noise to other parts of the building. Other rooms on this floor are a library, an X-ray room and a dark room.

The main feature of the top floor is the Conservation Department, containing about fifty chairs. The lighting is excellent and comes mainly from ceiling lights facing north. For dull days or evenings general illumination is obtained from large spherical opal lights, while a special light is provided for each chair. All advanced students will have the advantage of working with an up-to-date electrical unit.

A smaller room, containing ten chairs, for prosthetic and orthodontic dentistry is situated at the front of the building. There is also a demonstration theatre with a raised gallery for spectators and a special light which can be concentrated on to the mouth of the patient, thus allowing good visibility for the students watching an advanced procedure. The rest of the top floor is utilized by the Pathological Laboratory, a mannikin room where junior students can practise on dummies, and a room for the nursing staff.

The whole building is centrally heated by mechanically-stoked boilers in the basement. A car park and air raid shelter are adjacent.

The Hospital will, it is hoped, form a valuable addition to the existing medical services in the city, and will provide facilities for the training of students at least as good as those of other dental schools.

Now the problem of maintenance remains to be settled. The funds of the University can only be used for educational purposes, yet it must be anticipated that many indigent patients will receive treatment at the Dental Hospital. The cost of their treatment cannot lawfully be defrayed out of University funds. The Dental Hospital, like the other voluntary hospitals in our city, must be supported generously by charitable contributions, and perhaps to some extent by contributory schemes.

## Figures and Tables

**Figure f1:**
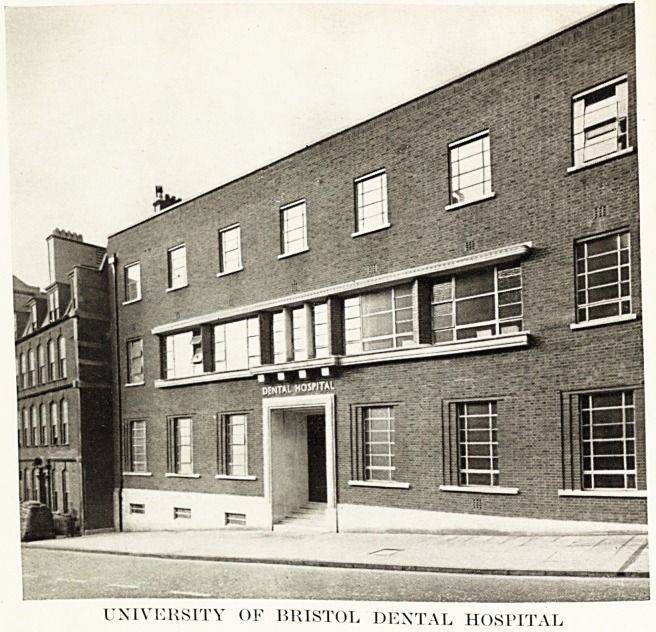


**Figure f2:**